# Functionality of hospital information systems: results from a survey of quality directors at Turkish hospitals

**DOI:** 10.1186/s12911-018-0581-2

**Published:** 2018-01-12

**Authors:** Mehmet Saluvan, Al Ozonoff

**Affiliations:** 1 Center for Applied Pediatric Quality Analytics, Boston Children’s Hospital, 300 Longwood Avenue, Boston, MA 02115 USA; 2 Department of Pediatrics, Harvard Medical School, Boston, MA USA

**Keywords:** Hospital information systems, Electronic health records, Healthcare quality, Patient safety, Health information technology

## Abstract

**Background:**

We aimed to determine availability of core Hospital Information Systems (HIS) functions implemented in Turkish hospitals and the perceived importance of these functions on quality and patient safety.

**Methods:**

We surveyed quality directors (QDs) at civilian hospitals in the nation of Turkey. Data were collected via web survey using an instrument with 50 items describing core functionality of HIS. We calculated mean availability of each function, mean and median values of perceived impact on quality, and we investigated the relationship between availability and perceived importance.

**Results:**

We received responses from 31% of eligible institutions, representing all major geographic regions of Turkey. Mean availability of 50 HIS functions was 65.6%, ranging from 19.6% to 97.4%. Mean importance score was 7.87 (on a 9-point scale) ranging from 7.13 to 8.41. Functions related to result management (89.3%) and decision support systems (52.2%) had the highest and lowest reported availability respectively. Availability and perceived importance were moderately correlated (*r* = 0.52).

**Conclusion:**

QDs report high importance of the HIS functions surveyed as they relate to quality and patient safety. Availability and perceived importance of HIS functions are generally correlated, with some interesting exceptions. These findings may inform future investments and guide policy changes within the Turkish healthcare system.

Financial incentives, regulations around certified HIS, revisions to accreditation manuals, and training interventions are all policies which will help integrate HIS functions to support quality and patient safety in Turkish hospitals.

**Electronic supplementary material:**

The online version of this article (10.1186/s12911-018-0581-2) contains supplementary material, which is available to authorized users.

## Background

There have been steady efforts to improve quality in healthcare since the early 2000s, kickstarted by two reports released by the Institute of Medicine (IOM) [1]. The first report asserts that healthcare is not as safe as it should be and offers a substantial body of evidence pointing to medical errors as a leading cause of death and injury in the United States (U.S.). The second report focuses more broadly on how the healthcare delivery system can be redesigned to innovate and improve care [2]. Both reports suggest making effective use of information technologies as one of six necessary strategies for the redesign of healthcare systems [2, 3] and express concern over slow uptake of information technology in healthcare. Healthcare is an information-based science [4] and providers must have access to timely and accurate information to provide safe high-quality care [5]. Clearly, information management and health information technology (HIT) are fundamental to current and future healthcare delivery in the U.S., [6, 7] United Kingdom (U.K.), [8] and elsewhere [9–15].

Modern healthcare makes wide use of information technology [16, 17]. Most stakeholders agree that information technology such as electronic health records (EHRs) and computerized provider order entry (CPOE) will be critical to transforming the healthcare industry [6]. According to the IOM, HIT must play a central role in the redesign of the healthcare system if a substantial improvement in quality is to be achieved over the coming decade. Given the complexity of modern medicine, it is inevitable that HIT will play an ever increasing role in improving healthcare quality [18]. The imperatives of improving documentation, reducing error, and empowering patients will continue to use of information technology in healthcare. There is plenty of evidence that clinical informatics applications can address these imperatives to enhance patient outcomes, reduced costs, and provide access to knowledge [19].

Otherwise, healthcare costs are rising and all parties involved-government, insurers, hospitals and patients-are concerned. Costs must be reduced, but without major compromise of quality [20, 21]. The widespread adoption of HIT may reduce costs by way of improved efficiency and less duplication of effort in delivery of care services as well as a reduction in costly medical errors [22, 23]. Payment systems and provisions from payers have further incentivized the use of information systems in healthcare [24]. For example, the Centers for Medicare and Medicaid Services (CMS) provide up to $27B of incentive payments over 10 years to hospitals and healthcare providers that demonstrate meaningful use of certified electronic health record (EHR) systems in the U.S. [25, 26]. Simply put, “meaningful use” requires providers to demonstrate use of HIT to measure improvements in quality of care [27]. Similarly, England has invested at least £12.8 billion in a National Programme for Information Technology for the National Health Service in 2009 [15, 28].

Hospitals in particular are characterized by the high capacity of information and clinical data produced, and a new category of HIS now dominates in modern hospitals [16]. These systems aim to support high-quality, efficient, patient-centered care [29] with integrated support for the administrative and management tasks needed to support such care [30]. HIS systems have been shown to decrease the cost of quality care and the accessibility time to patient records [24]. The relevance of ‘good’ HIS for high-quality of care is obvious [29, 30]. Further advances of technology in healthcare include the use of information and communication technology (ICT) to support robust communications in an increasingly complex healthcare environment. ICT originally contributed to timely and efficient transmission of patient data, and its focus is now shifting to improve clinical data quality by using online clinical data acquisition and processing [31].

Implementation of HIS^1^ systems has increased globally over the past 5 years, and higher-income countries are further in adoption and utilization of HIS systems compared to lower-income countries [32]. There are many competing HIS vendors each with their own products and different capabilities [33]. Most hospitals in higher-income countries are using comprehensive HIS, [12, 34–36] while in other parts of the world hospital orders for medications, laboratory tests, and other services are still paper-based [37]. This situation leads to a natural question: which core functions of HIS should be adopted for maximum impact on quality and patient safety?

This question was partially addressed in a 2003 IOM report which identified eight categories of core functionalities: health information and data; results management; computerized physician order entry; decision support system; electronic communication and connectivity; patient support; administrative processes; and reporting and population health management [38].

There is general consensus that the use of HIT should lead to more efficient, safer, and higher quality care [19, 39, 40]. There are few studies and data available on HIS implementation in countries with less mature healthcare systems. We hope to close this gap and provide new data on HIT implementation in Turkey.

Therefore the aim of this study is to determine availability of core HIS functions implemented in Turkish hospitals and their perceived importance on quality and patient safety.

## Methods

### Sampling frame

All licensed civilian hospitals in the nation of Turkey were eligible for this survey. Invitations to respond were sent to the Quality Director of each hospital from a listing of contact information maintained by the Turkish Ministry of Health (MoH). Military hospitals are not governed by the MoH and were excluded. In Turkish hospitals, QDs are responsible for planning and implementing quality and patient safety standards. Responsibilities of the QDs include: training and education of hospital staff; support and oversight of departmental quality committees; and coordination of internally and externally conducted audits [41]. We surveyed QDs since they are typically among the most knowledgeable staff about quality and patient safety aspects of hospital operations [42] (including use of HIS).

### Survey instrument

We developed a survey instrument to collect data and perceptions on core functions of HIS implementation in Turkey. In order to develop the survey items, we began with national and international hospital quality standards maintained by the MoH and Joint Commission International. We reviewed features of HIS with supporting evidence to facilitate hospitals meeting these quality standards. We supplemented this initial list of items with information from our review of the literature [38, 39, 41, 43, 44].

We note that two similar surveys have been conducted in the U.S. [39, 40]. Our instrument was developed independently for a specifically Turkish healthcare setting, but the items are derived from the same set of IOM documents and are broadly similar to the U.S. surveys. Davis and Thakkar delivered a brief 8-item survey to directors of Medical Informatics with three-level response scale (available/future implementation/no plans to implement) for each of the IOM-defined functionalities [40].We sought a more granular level of detail than that offered by this instrument. Jha et al. used a 32-item instrument, with each item reflecting one HIS function from 6 dimensions which approximate the IOM functionalities [39]. This instrument has been adapted for use in Japan, South Korea, and Spain [12, 14, 15]. Some items from the Jha survey are not appropriate for the Turkish setting, and we tailored the item selection to those most suitable to the Turkish healthcare system.

To assess the initial list of survey items, we performed a pilot study with a convenience sample of 17 QDs at hospitals across several provinces of Turkey. Staff roles in the pilot sample included physicians, nurses, computer engineers, healthcare administrators. The pilot survey was administered by email to the pilot study group and included 83 items. Each item described a core HIS function and used Likert scales to solicit opinions on the understandability and competency of each item to describe the intended HIS function. Many of these functions are either helpful or necessary to meet accreditation standards in the U.S. (e.g. by the Joint Commission), although there is no comparable national accreditation program implemented for hospitals in Turkey. Based on these pilot data, we updated the survey instrument by decreasing the number of items and revising some of the item descriptions. In particular, we removed or revised several items indicated as unclear or irrelevant based on free text comments from respondents.

The revised survey instrument includes 50 items, each of which describes a core functionality of HIS (see Additional file 1 for complete survey instrument translated from Turkish). Respondents are asked to provide two responses for each item. To reduce survey burden and complexity on respondents, we considered implementation to be ‘all or nothing’ and used binary responses to measure availability of core functions. The first scale measures availability of the HIS function with possible responses^2^: “available”, “not available”, or “unsure”. Responses of ‘unsure’ were excluded and we treated availability as a binary response for all analyses. The second scale measures perceived importance of each item on the quality of healthcare provided by the hospital. This was measured on a 9-point Likert scale, with an available response of “unsure”.

### Item classification

Each of the 50 items was classified into one or more of the IOM domains described as follows:

1. Health Information and Data (HID): HID functions deliver critical information to providers to make clinical decisions e.g. medical and nursing diagnoses, drug allergies, problem lists, and clinical narratives. If this information is unavailable, low-quality and inefficient care may result [38, 40].
2. Results Management (RM): RM functions manage electronically results of all types including laboratory test results, radiology procedure results, and pathology reports. Computerized results are more accessible, timely, and accurate [38].
3. Computerized Physician Order Entry (CPOE): CPOE applications transmit physician orders electronically to the appropriate clinical service units [45]. The benefits of CPOE include elimination of lost or duplicate orders, improved accuracy, and reduced time to fill orders.
4. Decision Support System (DSS): DSS provides clinicians, staff, patients, or other individuals with knowledge and person-specific information to enhance health and health care. It encompasses a variety of tools and interventions such as computerized alerts and reminders, clinical guidelines, order sets, patient data reports and dashboards, documentation templates, diagnostic support, and clinical workflow tools [46]. DSS applications are embedded in the HIS and aim to detect critical situations or errors in care, and then notify the clinician perhaps with additional information to assist with clinical decisions [4, 47].
5. Electronic Communication and Connectivity (ECC): ECC functions include electronic communication tools such as e-mail and web messaging. These systems have been shown effective in facilitating provider communication with other providers and with patients, allowing for improved continuity of care and more timely interventions [38, 48, 49].
6. Patient Support (PS): Patient support functions involve the usage of HIT to encourage participation in patient care of patients, patient families, or third party caregivers. PS functions include patient portal, recording and monitoring patient education provided by hospital staff [38, 40].
7. Administrative Processes (AP): AP functions include electronic scheduling systems for admissions, inpatient and outpatient procedures, and visits. These systems increase the efficiency of hospital administration and improve the patient experience [38, 45].
8. Reporting and Population Health Management (RPHM): RPHM functions provide public and private sector reporting at the federal, state, and local levels for safety, quality, and public health. This may include routine reporting of key quality indicators (sometimes referred to as clinical dashboards). This reduces the data collection and reporting burden, as well as the associated costs, and would likely increase the accuracy of the data reported [38, 50].

### Data collection and analysis

The survey was administered by email invitation and all data collected by web survey. Information and an external link to the survey were available on the Turkish MoH web portal accessible by the quality director or delegate at each civilian hospital in Turkey. Hospital QDs were also informed about the survey via two e-mail reminders during a two-week period in March 2015. By design, only one respondent at each hospital was permitted to submit responses.

The survey instrument contained 50 items as described above. There was a section available to record opinions and recommendations as free text (general evaluation section), and additional items on institutional, demographic, and professional characteristics including: geographic province and sector; hospital type and bed size; gender, age, educational level; job title, tenure in current hospital role, and total years of professional experience.

We calculated frequencies and percentages of respondent demographic and professional characteristics. For each HIS function, we calculated percentage of respondents indicating the function was ‘available’ and calculated the mean and median values of perceived impact on quality. We examined the bivariate relationship between percentage availability of each HIS function and mean perceived importance of that function using scatterplots and Pearsonian correlation analysis. We calculated mean percentage availability and importance scores averaged across all hospitals for each of the survey items (Table 3), and also averaged across items within each of the 8 IOM categories (Table 4).

We managed and analyzed data using the R statistical package (v3.1.0 2015 R Institute).

## Results

Institutional characteristics and response rates are reported in Table 1. 1486 hospitals were invited to participate, and we collected 464 responses (overall response rate 31.2% comparable to or greater than other similar national surveys [15, 40, 51]) representing all major geographic regions and 74 of 81 (91%) of provinces in Turkey. Response rates by section, hospital type, and bed size varied from 28.2% to 43.3%. Respondent hospital bed sizes ranged from 5 (minimum) to 1218 (maximum) beds (mean 157). The majority of responses came from general hospitals with fewer than 100 beds. Our sample does not differ greatly on key characteristics from eligible hospitals in Turkey in terms of sector (chi-square *p* = 0.22) or number of beds (*p* = 0.12), although the sample slightly under-represents general hospitals (77% of sample, 82% of eligible hospitals, *p* = 0.049).

**Table 1:** Institutional characteristics and response rates

	Eligible Hospitals in Turkey	Number of responses (% of sample)	Response rate (%)
Hospital Sector			
Private	542	189 (40.7)	34.9
Ministry of Health	874	252 (54.3)	28.8
University	70	23 (5.0)	32.9
Hospital Type			
Training Hospital	144	53 (11.4)	36.8
Specialty Hospital	120	52 (11.2)	43.3
General Hospital	1222	359 (77.4)	29.4
Number of Beds			
99 and below	973	274 (59.1)	28.2
100–199	241	88 (19.0)	36.5
200–299	88	30 (6.5)	34.1
300–399	46	15 (3.2)	32.6
400 and above	138	57 (12.3)	41.3
TOTAL	1486	464 (100.0)	31.2

The demographics and professional characteristics of the participants are shown in Table 2. The majority of respondent QDs were female, college educated, trained as nurses, under the age of 40*,* with fewer than 10 years professional experience and hospital tenure.

**Table 2:** Respondent demographics

Demographic features	Frequency (%)
Gender	
Female	314 (67.7)
Male	150 (32.3)
Education	
High school	18 (3.9)
Associate Degree	69 (14.9)
Bachelor Degree	211 (45.5)
Master	110 (23.7)
PhD	10 (2.2)
Medical Specialist	19 (4.1)
Missing	27 (5.8)
Job	
Physician	42 (9.1)
Nurse	235 (50.6)
Other Healthcare Staff	41 (8.8)
Engineer	11 (2.4)
Administrative Staff	101 (21.8)
Missing	34 (7.3)
Age Groups	
20–24	15 (3.2)
25–29	50 (10.8)
30–34	95 (20.5)
35–39	104 (22.4)
40–44	70 (15.1)
45–50	36 (7.8)
50 and above	22 (4.7)
Missing	72 (15.5)
Experience in current hospital work area/unit	
0–5 years	210 (45.3)
6–10 years	75 (16.2)
11–15 years	46 (9.9)
16–20 years	28 (6.0)
21–25 years	7 (1.5)
26 years and above	4 (0.9)
Missing	94 (20.3)
Experience in profession	
0–5 years	94 (20.3)
6–10 years	78 (16.8)
11–15 years	59 (12.7)
16–20 years	53 (11.4)
21–25 years	45 (9.7)
26 years and above	32 (6.9)
Missing	103 (22.2)

Availability and mean perceived importance are reported for all 50 items (Table 3). Mean perceived importance was 7.87 (SD 1.71). Across all items, we observed lowest and highest means respectively of 7.13 (SD 2.25) (Item 2: predict time to examination on admission) and 8.41 (SD 1.81) (Item 50: data security). Availability of HIS functions, averaged across all 50 items and across all hospitals, was 65.6% (SD 20.0), with availability on particular functions ranging from 19.6% (SD 39.5) (item 15: telemedicine applications) to 97.4% (SD 16.0) (item 49: authorized access for staff). Respondent QDs reported that all HIS functions surveyed have an important effect on quality and patient safety (mean 7.87 on 9-point scale, SD 1.71).

**Table 3:** Average of availability and percieved importance [mean (SD)]

Item No	HIS functions	Availability (%)	Perceived importance (Over 9)
1	Display alerts for high risk medications (e.g. narcotics, sound-alike drugs, concentrated electrolytes) just prior to administration	47.7 (50.0)	8.07 (2.05)
2	Predict time to patient examination on admission	74.6 (43.6)	7.13 (2.25)
3	Record time to consultation after request from emergency department	82.4 (38.2)	7.66 (2.13)
4	Display real-time availability of patient beds	91.9 (27.4)	7.84 (1.99)
5	Flag and prioritize elderly and disabled patients	80.8 (39.4)	7.81 (1.99)
6	Provide electronic copy of patient records when requested (e.g. diagnosis list, lab test results, administered procedures, administered medications, discharge summary)	82.9 (37.7)	7.58 (2.02)
7	View all diagnostic test results including laboratory, radiology, pathology, nuclear medicine, endoscopy	92.4 (26.5)	8.12 (1.85)
8	Record times and performing staff of laboratory test samples throughout all laboratory phases i.e. sampling, accepting, analyzing, approving, and reporting	96.1 (19.4)	8.19 (1.79)
9	Display alerts for lab samples that do not meet acceptance criteria	72.4 (44.8)	7.96 (1.98)
10	Display alerts for laboratory tests that return panic values	96.5 (18.4)	8.33 (1.79)
11	Display reminders for internal and external lab quality control measures and keep their result for analyses	57.8 (49.4)	7.65 (2.12)
12	Record laboratory process problems (pre-analytic, analytic, and post-analytic)	71.0 (45.4)	7.76 (2.02)
13	Provide digital radiology images within internal network i.e. Picture Archiving and Communication System (PACS)	85.3 (35.5)	8.18 (1.89)
14	Monitor radiology appointment and reporting times	61.1 (48.8)	7.46 (2.13)
15	Support telemedicine applications	19.6 (39.5)	7.58 (2.28)
16	Monitor use of blood and blood products during order, preparation, acceptance, and implementation	74.3 (43.8)	8.11 (1.90)
17	Monitor blood and blood products stock and expiration date	78.1 (41.4)	8.16 (1.91)
18	Record disease severity as structured data (e.G. *apache* II, SAPS II, and PRISM)	50.7 (50.1)	7.64 (2.10)
19	Integrate nursing care plans into medical record	58.5 (49.3)	7.57 (2.26)
20	Record and integrate all clinical orders into medical record including laboratory test orders, medication orders, nursing care orders, nutrition therapy orders, rehabilitation therapy orders	84.2 (36.5)	7.94 (1.99)
21	Record and integrate all diagnostic, clinical, and surgical procedures into medical record including endoscopy, cardiac catheterization, radiotherapy, CT, and ultrasound	90.1 (29.9)	8.05 (1.95)
22	Record usage and monitor complications of anesthetic agents and sedatives administered outside of anesthesiology (e.g. endoscopy, cardiac catheterization, and IVF units)	52.2 (50.0)	7.81 (2.08)
23	Display clinical guidelines and provide alerts for deviations	50.2 (50.1)	7.53 (2.10)
24	Provide alerts for drug-drug interactions	45.5 (49.9)	7.98 (2.06)
25	Provide alerts for drug-food interactions	42.3 (49.5)	7.88 (2.12)
26	Provide alerts for drug-allergy interactions	41.9 (49.4)	8.07 (2.06)
27	Integrate Computerized Physician Order Entry	75.6 (43.0)	8.04 (1.98)
28	Provide alerts for patient education that is part of care plan or discharge plan	29.7 (45.8)	7.39 (2.23)
29	Record all patient education provided to patient	41.8 (49.4)	7.32 (2.18)
30	Record patient and staff safety events	57.0 (49.6)	7.86 (2.11)
31	Record emergency code alerts (e.g. Code Blue, Code White) and integrate with paging system	42.6 (49.5)	7.67 (2.22)
32	Record blood transfusion reactions as structured data	52.0 (50.0)	7.84 (2.17)
33	Monitor hospital key performance indicators automatically	62.6 (48.5)	7.96 (2.00)
34	Flag patients and warn staff of patients with risk of infection (e.g. HIV+, HepC+)	51.7 (50.0)	8.18 (2.04)
35	Monitor nosocomial infections and transmit surveillance data to national or international networks	44.8 (49.8)	7.83 (2.13)
36	Monitor and record sterilization processes including procedure date and time and material expiration date	32.8 (47.0)	7.76 (2.19)
37	Display real-time information necessary for unit managers including bed occupancy, waiting list, surgeries scheduled	88.9 (31.5)	7.85 (2.01)
38	Report periodically on operational statistics including monthly admissions, income-expenditure, services delivered	94.5 (22.8)	7.88 (1.94)
39	Provide alerts for devices that require periodic maintenance or calibration	29.5 (45.7)	7.76 (2.11)
40	Provide alerts for medications and medical supplies near expiration or at critically low stock levels	91.9 (27.3)	8.25 (1.85)
41	Provide inventory of all medical devices including location and responsible staff	57.4 (49.5)	7.65 (2.08)
42	Monitor and provide reminders for routine health screening of clinical staff	25.6 (43.7)	7.80 (2.13)
43	Identify, flag, and, prohibit duplicate patient records	83.0 (37.6)	8.14 (1.95)
44	Monitor occupational accidents and injuries	52.0 (50.5)	7.90 (2.10)
45	Display current organizational policies and procedures and provide alerts of updated documents	67.5 (46.9)	7.90 (2.09)
46	Record clinical staff certification and licensing information	78.8 (40.9)	7.80 (1.97)
47	Integrate and update information with hospital external website	56.1 (49.7)	7.73 (2.10)
48	Provide online patient portal to view, download, and transmit lab results	91.1 (28.5)	8.09 (1.86)
49	Provide access control management for different staff groupings	97.4 (16.0)	8.18 (1.85)
50	Provide data security and protection for electronic health information	93.6 (24.5)	8.41 (1.81)
	**Average**	65.6 (20.0)	7.87 (1.71)

**Fig. 1:**
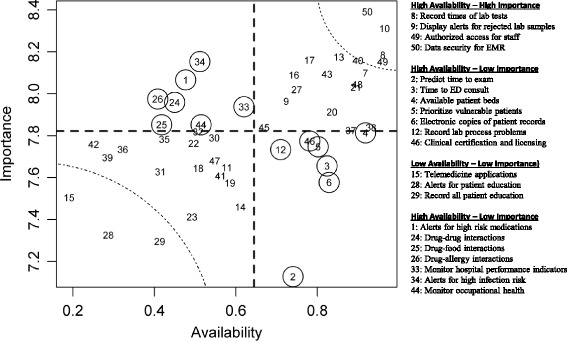
Relationship of availability and perceived importance for each of 50 HIS functions. This figure illustrates the bivariate relationship across all hospitals surveyed between availability (%) and perceived importance (mean) for each of 50 HIS functions. Items of special interest are enclosed within circles or dotted regions

We plotted the bivariate relationship between availability and perceived importance of HIS functions (Fig. 1). Availability and mean perceived importance are moderately correlated (*r* = 0.52, *p* = 0.0001). Functions with highest availability and perceived importance were those related to data security (items 49, 50) and laboratory services (items 8, 10), while those with lowest availability and perceived importance related to telemedicine (item 15) and patient education (items 28, 29). Functions related to staff safety (items 34 and 44), medication safety (items 1, 24, 25, 26), and monitoring indicators (item 33) were less available than expected relative to their perceived importance. Conversely, functions related to patient access, comfort, and rights (items 2, 3, 5, 6) and operational activities (items 4, 12, 46) had lower perceived importance than we would expect based on availability.

We categorized all 50 items into eight non-exclusive IOM categories of HIT functionality (Additional file 1) and ranked the availability of functions within these domains from highest to lowest (Table 4). Functions related with result management (89.3%) and decision support systems (52.2%) had the highest and lowest reported availability score respectively. Respondent QDs reported functions related with CPOE to have the highest importance in terms of improving healthcare quality, while functions related with patient support were ranked as lowest.

**Table 4:** Availability and perceived importance by IOM classification [mean (SD)]

	Availability (%)	Perceived importance
Result Management	89.3 (18.5)	7.87 (1.72)
Administrative Processes	71.7 (18.7)	7.86 (1.71)
Computerized Physician Order Entry	68.0 (25.1)	7.93 (1.80)
Electronic Communication and Connectivity	64.2 (21.3)	7.87 (1.72)
Reporting and Population Health Management	63.0 (24.5)	7.79 (1.78)
Health Information and Data	62.6 (24.0)	7.89 (1.75)
Patient Support	62.5 (27.1)	7.67 (1.76)
Decision Support Systems	52.2 (28.8)	7.88 (1.79)

Free text comments are classified and sorted by frequency (Table 5). Many respondents highlighted the importance of the study, their desire to review HIS from the perspective of quality and patient safety, and higher expectations of HIS functions to improve quality and patient safety.

**Table 5:** Comments collected from the “general evaluation” section

Participant comments	Frequency
Thanks for the study and for the opportunity to evaluate HIS functionalities currently in use	33
Every HIS function in survey would positively affect quality of healthcare	22
Some functions are available as a part of our HIS, but they are not used	14
Turkish MoH should produce and share HIS for common use	12
HIT department support is insufficient, ineffective, or disregards users; technical problems lead to sub-optimal use	12
Every HIS function in survey should be mandatory for every HIS	10
Employees and managers lack training and experience to use HIS effectively	7
MoH should define certification standards for HIS and related products	5
Comments about specific functions (PACS, telemedicine, monitoring indicators, nursing care plans)	5
Suggested HIS functions related to quality and patient safety not included in survey	5
National integration of HIS and patient records available to all healthcare institutions	3
Integration problems between information systems used by MoH and hospitals	3
Total	**131**

## Discussion

We investigated availability of HIS functions and their perceived importance on quality and patient safety by 50 items survey developed by authors, and received responses from hospital quality professionals from a broadly representative sample of Turkish hospitals in terms of geographic and institutional characteristics. Despite high levels of perceived importance across all 50 HIS functions which comprised our survey, on average only two-thirds of hospitals surveyed have adopted these functions and important functions like decision support systems are adopted at very low rates.

We focused on subgroups of HIS functions that are of special interest. First we considered the more highly available functions. Generally the most widely available functions are those related to data security, automation of laboratory processes, and administrative reporting. Reasons for this high adoption may include the importance of privacy and data security in the healthcare setting [52, 53]; pressure on laboratories to satisfy ongoing quality audits and maintain licensing or certification; or recommendations from MoH [54–56]. It is also worth noting that administrative functions are often highly valued by hospital leaders who are in a position to influence HIS purchasing decisions [6, 45, 57].

We examined more closely the relationship between availability and perceived importance of each item. We observed as expected that availability and perceived importance are generally correlated (Fig. 1). However there were some functions that departed from the general pattern. For example, we identified three ‘clusters’ of items which were generally not as available as their perceived importance would suggest: staff safety, medication safety, and monitoring indicators. While focus has recently shifted to patient-centered care, it is also increasingly recognized that high quality care includes employee safety as well as patient safety. This change of emphasis is meant to ensure that a safety culture pervades a healthcare organization, with the safety of the workforce and the work environment given equal standing as a safety priority [58]. Medication errors compose a sizable proportion of the total burden of medical errors, and information systems are an effective tool to prevent these errors [2, 3, 59, 60]. Monitoring indicators in real-time using HIS offers many advantages [61, 62]. This reflects discordance between hospital QDs and the hospital leaders who make decisions about HIS purchasing.

Functions associated with patient access to services, patient comfort, patient rights, and hospital operations were evaluated as relatively less important than their availability. Indeed, QDs are expected to be more sensitive to patient satisfaction and patient experience. However our findings suggest that the administrator responsible for HIS selection might prioritize administrative needs and patient expectations driven by previously determined health policies [63] that might lead to diminished perceived importance among healthcare staff include quality directors.

Availability and perceived importance were concordant and generally high for features related to information security, patient safety, and laboratory services. Information security is a top priority for every institution and especially for hospitals, although there are no privacy laws in Turkey specific to healthcare such as the Health Information Portability Accountability Act (HIPAA). Moreover, patient safety and laboratory services standards were component of national quality standards from the first iteration, and hospitals have been surveyed against these standards several times [54, 64]. These findings are consistent with a positive effect from the quality survey process explained above.

Telemedicine applications and patient education functions had generally low availability and perceived importance. Although telemedicine has significant advantages for patients located in remote geographic regions far from qualified healthcare facilities [65–68], our finding is not surprising since many hospitals without remotely located patients may consider telemedicine a luxury. Patient empowerment via education is understood to be an important part of the healing process [69, 70]. We believe our findings have identified an important issue for further discussion among healthcare administrators and employees.

The IOM category reported most available was RM, with these functions present on average across 90% of respondent hospitals. Managing results electronically has substantial impact on unnecessarily ordered tests, and timeliness of reporting to providers. It is also significant to prevent medical errors [71–73]. The high level of RM function adoption among Turkish hospitals may accelerate future plans for electronic medical records shared throughout all Turkish healthcare providers, leading to eventual improvements in clinical care by increasing provider access to timely and accurate clinical data. Conversely, DSS is reported as the functional category least available in Turkish hospitals. DSS includes applications that combine clinical information with embedded medical knowledge to assist the human decision process [4]. These systems imply some higher level of information processing, or inference, by the computer [74]. Some respondents reported that both providers and hospital managers lacked the necessary knowledge for effective use of information systems (See Table 5). Thus general issues of staff training and expertise of management may explain lower availability of DSS in Turkish hospitals, as well as clinicians’ willingness to ask, direct, and help vendors and hospital managers with the development and adoption of DSS. The literature supports the potential of DSS to improve patient safety and quality of care e.g. reminders for vital tasks, assistance with diagnoses, avoiding drug-drug interactions, enhancing clinical regulatory compliance, reducing unnecessary test orders, and identifying emerging disease outbreaks [6, 74–76]. Involving clinicians in DSS development, and increased awareness and investment in DSS will likely improve safety and quality across the Turkish hospital system.

Perceived importance was generally high across all items, and there were no substantial differences in across IOM categories. CPOE functions were perceived as most important and PS functions (e.g. patient portal) were generally perceived as less important. Similar research studying 89 U.S. healthcare facilities in 2005 rated categories 7.58 to 8.83 (out of 10) on importance, similar to our results. PS shows low in the U.S. as well as Turkey [40]. The same study showed 37% of respondents report current use of at least one component in all of the eight core HIS functionalities, while another study suggests that in 2009, only 1.5% of U.S. hospitals had comprehensive HIS i.e. present in all clinical units, and only an additional 7.6% have a basic system i.e. present in at least one clinical unit. Results from the U.S., Spain, and South Korea also show highest availability for RM functionality e.g. RM was available for 75% of U.S. hospitals [14, 15, 39]. There are broad parallels between the U.S. situation circa 2010 and the adoption of HIS in Turkey roughly 5 years later.

This study has several limitations. Although we examined reliability of individual items, we did not validate our survey using an independent sample nor did we validate against an existing instrument. We designed and piloted our survey with an aim of face validity, and our focus was on broad patterns of response rather than the psychometric properties of the survey instrument. The survey instrument does not include all HIS functions but rather 50 items to be most relevant to patient safety and quality as determined by researcher judgment and pilot testing. Although observed scores for perceived importance lie within a compressed range of values – mean responses across items range from 7.13 to 8.44 – our large sample allows for valid statistical comparison and correlation. Military hospitals were not included in the sample. Finally, promotion of the survey via the MoH e-mail and website may have induced a ‘halo effect’ of positive response bias.

Diverse HIS functions have been adopted at different rates in Turkey and across the world. This study, which supports previous research conducted in the U.S., presents potentially useful insight into the adoption of HIS functions to support patient safety and quality. Findings from this and similar surveys should be considered carefully by policymakers, software designers, clinicians, and hospital leaders. We recommend mandating certified software (or offering incentives) and surveying HIS functions during regular quality audits, two policy approaches which would support system-wide improvements. Another important issue that we identified is the training of hospital leaders and clinical staff. Technical support and training processes may be better defined by public regional authorities. Software designers may consider our results when developing HIS products and hospital leaders should make HIS purchasing decisions using information from QDs on which functions are perceived most important to patient safety and quality of care.

## Conclusions

Our study corroborates previous work highlighting the perceived importance of HIT on quality and patient safety. After revision and tailoring to the specifics of other international settings, the expanded list of items in this study could be used elsewhere to increase awareness and to survey availability of HIS functions in other national healthcare systems. We believe that our survey is an important first step to understand the system-wide availability of specific HIS functions across hospitals in Turkey, and that similar surveys in other countries would yield valuable knowledge to guide policymakers and hospital leaders in many settings.

Our findings support the conclusion that HIS functions in Turkish hospitals are generally not as available as quality managers would like. Policymakers, hospital leaders, and software developers all have a potential role to address future improvements. Some policy levers include financial incentives to adopt specific HIS functions; government involvement in certification of software; regulations to encourage or enforce usage of certified HIS; and inclusion of desired functions into accreditation manuals. Each of these policies may help integrate HIS functions to support quality and patient safety in Turkish hospitals. Finally, further investment in training programs will be needed across organizational levels, including clinical employees, HIT support staff, and hospital leaders and managers.

## Additional file

Additional file 1: Survey Items Related to IOM Groups. This table provides classification of survey items within the 8 HIS functionality categories described by IOM. (XLSX 13 kb)

## Abbreviations

AP: Administrative Processes
APACHE: Acute physiology and chronic health evaluation
CMS: Centers for Medicare and Medicaid Services
CPOE: Computerized physician order entry
CT: Computed tomography
DSS: Decision support system
ECC: Electronic communication and connectivity
EHR: Electronic health records
EMR: Electronic medical record
HepC: Hepatitis C
HID: Health information and data
HIPAA: Health Information Portability Accountability Act
HIS: Hospital information systems
HIT: Health information technology
HIV: Human immunodeficiency virus
ICT: Information and communication technology
IOM: Institute of Medicine
IVF: In vitro fertilization
MoH: Ministry of Health
PACS: Picture Archiving And Communication System
PRISM: Pediatric risk of mortality
PS: Patient support
QDs: Quality directors
RM: Result management
RPHM: Reporting and population health management
SAPS: Simplified acute physiology score
SD: Standard deviation
U.K.: United Kingdom
U.S.: United States

## Footnotes

1 Terms such as Hospital Information System (HIS), Electronic Medical Record (EMR), Electronic Health Record (EHR), and Patient Health Record (PHR) are used interchangeably in the literature. We have tried to use the term Hospital Information System (HIS) consistently throughout this paper unless it is necessary or relevant to differentiate terms.

2 These terms are translated from Turkish responses “var”, “yok”, and “fikrim yok”.
